# Chemical Profiling and Biological Evaluation of *Nepeta baytopii* Extracts and Essential Oil: An Endemic Plant from Turkey

**DOI:** 10.3390/plants10061176

**Published:** 2021-06-09

**Authors:** Gokhan Zengin, Mohamad Fawzi Mahomoodally, Abdurrahman Aktumsek, József Jekő, Zoltán Cziáky, Maria João Rodrigues, Luisa Custodio, Rıdvan Polat, Ugur Cakilcioglu, Adnan Ayna, Monica Gallo, Domenico Montesano, Carene Picot-Allain

**Affiliations:** 1 Physiology and Biochemistry Research Laboratory, Department of Biology, Science Faculty, Selcuk University, Campus, Konya 42130, Turkey; gokhanzengin@selcuk.edu.tr (G.Z.); aktumsek@selcuk.edu.tr (A.A.); 2 Department of Health Sciences, Faculty of Medicine and Health Sciences, University of Mauritius, Réduit 80837, Mauritius; f.mahomoodally@uom.ac.mu (M.F.M.); picotcarene@yahoo.com (C.P.-A.); 3 Agricultural and Molecular Research and Service Institute, University of Nyíregyháza, 4405 Nyíregyháza, Hungary; jjozsi@gmail.com (J.J.); cziaky.zoltan@nye.hu (Z.C.); 4 Centre of Marine Sciences, Faculty of Sciences and Technology, University of Algarve, Ed. 7, Campus of Gambelas, 8005-139 Faro, Portugal; mary_p@sapo.pt (M.J.R.); lcustodio@ualg.pt (L.C.); 5 Department of Landscape Architecture, Faculty of Agriculture, Bingol University, Bingöl 12000, Turkey; rpolat@bingol.edu.tr; 6 Department of Botany, Pertek Sakine Genç Vocational School, Munzur University, Tunceli 62000, Turkey; ucakilcioglu@yahoo.com; 7 Department of Chemistry, Faculty of Sciences and Arts, Bingol University, Bingöl 12000, Turkey; aayna@bingol.edu.tr; 8 Department of Molecular Medicine and Medical Biotechnology, University of Naples Federico II, via Pansini, 5, 80131 Naples, Italy; 9 Department of Pharmacy, University of Naples Federico II, via D. Montesano 49, 80131 Naples, Italy

**Keywords:** *Nepeta*, polyphenols, antioxidant, enzyme inhibition, phytopharmaceutics

## Abstract

*Nepeta baytopii* is a poorly studied, endemic *Nepeta* species (*Lamiaceae*) of Turkey. For the first time, the biological activities (antioxidant, enzyme inhibition, and cytotoxicity properties) of the hexane, ethyl acetate, methanol, water/methanol, and water extracts and essential oil prepared from *N. baytopii* aerial parts were assessed. Hydro-methanol (41.25 mg gallic acid equivalent (GAE)/g) and water extracts (50.30 mg GAE/g), respectively showed the highest radical scavenging (94.40 and 129.22 mg Trolox equivalent (TE)/g, for 2,2-diphenyl-1-picrylhydrazyl radical and 2,2-azino-bis (3-ethylbenzothiazoline-6-sulfonic acid radical scavenging assays) and reducing (229.37 and 129.55 mg TE/g, for ferric-reducing antioxidant power and cupric-reducing antioxidant capacity assays) capacities in vitro. An interestingly high inhibition was observed for ethyl acetate extract against butyrylcholinesterase (10.85 mg galantamine equivalent/g). The methanol extract showed high cytotoxicity (31.7%) against HepG2 cells. Caryophyllene oxide was identified in high concentrations in the essential oil (39.3%). Luteolin and apigenin and their derivatives were identified from the methanol and water extracts. The results obtained from this study highlighted that the abundance of highly bioactive compounds from *Nepeta baytopii* ensures the multiple biological activities of the tested extracts, and this suggests a potential use in the pharmaceutical and nutraceutical fields, and therefore should be investigated further.

## 1. Introduction

Nowadays, humanity faces several problems, including both infectious and non-infectious diseases. The prevalence of some non-infectious diseases, such as Alzheimer’s disease, diabetes mellitus, or obesity, is globally increasing by the day, and urgent precautions are needed to combat these diseases. Considering the increasing human population, synthetic precautions are still the most common for managing this fact. However, most synthetics have exhibited unfavorable side effects on human health, and we have to change them to safe, natural ones. In this sense, phytochemicals are considered in the natural arsenal for humanity [1,2,3]. Phytochemicals, including phenols, phenolic acids, flavonoids, tannins, and terpenoids, amongst others, are secondary metabolites possessing biological activities [4,5,6]. For decades now, humankind has been studying the intricate composition of plant extracts to harness their biological activities. Species of the *Lamiaceae* family possess therapeutic activity regarding gastroenterology, dermatology, and gynecology, and the herbs and leaves of *Lamiaceae* species have been used to treat respiratory complications [7]. The *Lamiaceae* family is a family of flowering plants, consisting of 236 genera composed of 6900–7200 species, and *Nepeta* is one of the largest genera of this family [7]. With some 280 species, the *Nepeta* genus is distributed over central and southern Europe, and western, central, and southern Asia, and North Africa [8].

The distinctive diversity and richness of regions of southwestern Asia, including Turkey and Iran, makes it a hotspot of the *Nepeta* genus [9]. In Turkey, 33 *Nepeta* species have been recorded, and 17 of them are endemic [9]. *Nepeta* species have been used in traditional medicine for their antiseptic, antispasmodic, anti-asthmatic, febrifuge, antitussive, and diuretic properties [8]. Additionally, in Turkey, the members of the *Nepeta* genus have been widely used for colds, cancers, coughing, rheumatism, wound healing, obesity, and stomachaches [10,11,12,13]. In addition, a recent comprehensive review published by Shara and colleagues [14] presented the in vivo and in vitro studies reporting the acetylcholinesterase inhibitory, anti-atherosclerotic, anticonvulsant and myorelaxant, antidiabetic, anti-leishmanial, anti-malarial, anti-melanogenesis, antioxidant, anthelmintic, hepatoprotective, cytotoxic, immunomodulatory, cardioprotective, anti-microbial species of the *Nepeta* genus. Salehi et al. [15] reviewed the presence of significant compounds in the *Nepeta* genus, including nepetalactone, β-caryophyllene, germacrene-D, 1,8-cineole, and α-pinene.

The biological activities of some *Nepeta* species endemic to Turkey, namely, *Nepeta italica* subsp. *cadmea*, *N. nuda* subsp. *glandulifera*, *N. meyeri, N. conferta,* and *N. cadmea* have been previously investigated [8,9,16,17,18,19]. However, endemic *Nepeta baytopii* has received little scientific attention. In an earlier study conducted by Dirmenci et al. [20], the morphological description and threatened categories of four *Nepeta* species were reported. In their study, *N. baytopii* were described as perennial with a height of 25–70 cm. The plant has longer-spreading hairs and sessile glands. Leaves are ovate-triangular, and the colour of the corolla is lilac. In the paper, the plant is described as an endangered species based on IUCN categories. In another study performed by Kılıc et al. [21], three *Nepeta* species were investigated for determining the essential oil composition, and one of the species was *N. baytopii*. In light of the above-mentioned point, this study was designed to provide additional data and novel insights on *N. baytopii.* In this sense, this work focuses on the evaluation of the hexane, ethyl acetate, methanol, water/methanol, and water extracts and essential oil prepared from *N. baytopii* aerial parts. Biological properties, namely, antioxidant and enzyme-inhibitory properties of all extracts and essential oil, were determined by using in vitro spectrophotometric methods. To determine the cytotoxic effects of the methanol and water extracts, three cell lines (HepG2, B16A5 and S 17) were used. Moreover, methanol and water extracts and essential oils were chemically characterised by using chromatographic methods. It is anticipated that gathered data herein will contribute towards establishing baseline data on this endemic species with significant medicinal potential.

## 2. Results and Discussion

Folin–Ciocalteu and aluminium chloride assays are rapid, simple, and low-cost procedures which provide an overview of the phenolic and flavonoid contents of plant extracts. These widely used methods underpin detailed phytochemical profiling using cutting-edge technologies. As shown in Table 1, the water extract (50.30 mg GAE/g) possessed the highest concentration of phenolics, followed by the water/methanol extract (41.25 mg GAE/g). The ethyl acetate extract rich in flavonoids contained the value of 27.02 mg RE/g. Hexane extract possessed the lowest phenolic (13.23 mg GAE/g) and flavonoid (7.77 mg RE/g) contents. Additionally, both total amounts of phenolics and flavonoids were significantly affected by the extraction solvents used (*p* < 0.05). This observation was also confirmed by several authors, who reported that the used solvents affected the level of phenolics and flavonoids of *Nepeta* extracts [16,22]. Detailed profiles of *N. baytopii* aerial parts methanol and water extracts were provided in Table 2 and Table 3, respectively. UHPLC profiling confirmed the presence of 46 and 43 compounds from methanol and water extracts, respectively. Chromatograms are depicted in Supplemental Material (Figures S1 and S2). Flavones, such as luteolin and apigenin and their derivatives, were identified from both extracts. Fertaric acid, a hydroxycinnamic acid and ester of ferulic acid and tartaric acid, was present in the methanol and water extracts. In general, the detailed phytochemicals of the methanol and water extracts of *N. baytopii* aerial parts were quite similar, which might be related to the polar nature of the solvents. In addition, the concentration of the different components in the methanol and water extracts might be different, and this was not specified in our data. In accordance with our results, the presence of flavones and hydroxycinnamic acid in the members of the *Nepeta* genus was reported in earlier studies [23,24,25,26,27].

GC-MS was used to determine the composition of *N. baytopii* aerial parts in the essential oil, and the data were presented in Table 3. A total of 10 compounds have been identified from the *N. baytopii* essential oil. Caryophyllene oxide, a sesquiterpenoid oxide common to lemon balm and eucalyptus, was identified in high concentration in *N. baytopii* essential oil (39.3%) [28]. Another sesquiterpene, spathulenol (15.6%), was identified in appreciable amounts from *N. baytopii* essential oil. Kilic and colleagues also reported a lower concentration of caryophyllene oxide in the essential oil of *N. baytopii* aerial part [21]. These differences in the levels of essential oil components could be explained by geographical and climatic differences. Additionally, different compounds (caryophyllene, limonene, nepetalactone and 1,8-cineole, etc.) were identified as main components in the essential oils of some *Nepeta* species [9,29,30,31].

**Figure 1:**
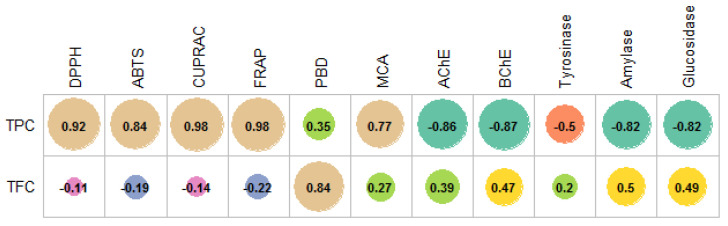
Pearson’s correlation between total bioactive compounds and antioxidant properties and enzyme inhibition effects (*p* < 0.05).

The dearth of scientific information regarding the antioxidant capacities of *N. baytopii* has fuelled the need for the comprehensive evaluation of the antioxidant properties of the different extracts and the essential oil of this endemic species. In order to evaluate the antioxidant properties of the extracts and essential oil of *N. baytopii* aerial part, six bioassays were conducted. These assays included free-radical scavenging (DPPH and ABTS), reducing power (FRAP and CUPRAC), metal chelating, and phosphomolybdenum. These findings are presented in Table 4. Several studies have reported the relationship between high phenolic/flavonoid content and antioxidant activity [32,33,34]. The total antioxidant capacity of the extracts was assessed using the phosphomolybdenum method. As shown in Table 4, the methanol (2.45 mmol TE/g) and ethyl acetate (2.36 mmol TE/g) extracts were the most active. However, we did not observe any statistical difference among the ethyl acetate and methanol extracts (*p* > 0.05). Moreover, essential oil *N. baytopii* aerial parts also showed a better total antioxidant ability than those of water/methanol and n-hexane extracts (*p* < 0.05). The total antioxidant ability could be attributed to the presence of different compounds in the extracts or essential oils. In this sense, as can be seen in Figure 1, we observed a weak correlation between total phenolics and phosphomolybdenum results, but the correlation value was high for total flavonoids. Determining the ability of natural compounds to quench free radicals provides an estimation of their possible scavenging activity in other systems. As shown in Table 4, the water/methanol extract of *N. baytopii* aerial part showed the highest scavenging activity against DPPH (94.40 mg TE/g) and ABTS (129.22 mg TE/g). In the ABTS assay, n-hexane, ethyl acetate, and the essential oil exhibited similar scavenging abilities (*p* > 0.05). In addition, these extracts and the essential oil did not have any scavenging ability on the DPPH radical. The reducing capacity of the compounds to donate an electron and thus act as reducing agents is commonly assessed using two widely used methods, namely, FRAP (ferric ion) and CUPRAC (cupric ion) assays [35]. In the present study, the water extract of *N. baytopii* exhibited the highest Fe^3+^ (129.55 mg TE/g)- and Cu^2+^ (229.37 mg TE/g)-reducing potentials. In these reduced power assays, all tested samples exhibited different abilities (*p* < 0.05). As can be seen in Table 1 and Table 5, generally, the free radical scavenging and reduced power results could be correlated with their total phenolic contents (*r* > 0.8). Pearson’s correlation coefficient values are given in Figure 1. Thus, it resulted that phenolic compounds are the main contributors to the antioxidant properties of *N. baytopii.* Similarly, several researchers have reported a strong correlation between antioxidant properties and the total amounts of phenolics [32,36,37]. Moreover, some authors have argued that the phenolic compounds in the members of the *Nepeta* genus were main players in the antioxidant assays [30,38,39]. As another mechanism, transition metals are known to participate in Fenton reactions, generating free radicals and exacerbating the oxidative stress status. Therefore, the chelation capacity of *N. baytopii* aerial parts extracts and essential oil were assessed. Results presented herein demonstrated that the water extract and water/methanol extracts possessed a stronger chelating ability as compared with other extracts and essential oils (*p* < 0.05). The metal-chelating abilities of the tested extracts might be due to the presence of phenolics, and the correlation analysis was confirmed by this fact (*r* = 0.77).

The inhibitory ability of *N. baytopii* aerial parts extracts and essential oil were tested against enzymes linked to a critical role in the development of diabetes mellitus type II, Alzheimer’s disease, and skin hyperpigmentation problems. Diabetes mellitus type II and Alzheimer’s disease have escalated to epidemic proportions, and the need for complementary therapeutic agents to effectively manage these debilitating conditions are of paramount importance. From Table 5, the ethyl acetate extract of *N. baytopii* aerial parts exhibited the highest activity against AChE (4.57 mg GALAE/g) and BChE (10.85 mg GALAE/g). In AChE inhibition, n-hexane and methanol extracts displayed similar actions (*p* > 0.05). The high galantamine equivalent value recorded on BChE supported appreciably high inhibitory action in comparison to other *Lamiaceae* species [40,41,42]. The inhibition of BChE has been advocated in the later stage of Alzheimer’s disease. During the progression of the disease, the BChE level increases, exacerbating the conditions of the patient [43]. Herein, the ability of *N. baytopii* aerial parts extracts and essential oil to inhibit α-amylase and α-glucosidase was also evaluated. These enzymes play critical roles in hyperglycaemia, the hallmark of diabetes mellitus. In diabetes mellitus type II management, the inhibition of enzymes responsible for the hydrolysis of polysaccharides to glucose monosaccharide, which can be absorbed in the intestinal system. Herein, methanol extract showed the highest (8.15 mmol ACAE/g) activity against α-glucosidase. Interestingly, the hexane and ethyl acetate extract were also good inhibitors of α-glucosidase. α-Glucosidase situated at the brush border of the small intestine catalyses the hydrolysis of disaccharides into glucose. Therefore, the inhibition of α-glucosidase reduces glucose formation, glycaemic peaks, and hyperglycaemia. Apart from these debilitating maladies, the ability of *N. baytopii* aerial parts extracts and essential oil to inhibit tyrosinase was also evaluated. Tyrosinase is the key enzyme targeted in skin hyperpigmentation treatment. In fact, the inhibition of tyrosinase reduces the production of the brown pigment melanin. Herein, the methanol and water/methanol extracts possessed the highest tyrosinase inhibition values (*p* > 0.05). The search for natural compounds possessing tyrosinase inhibitory characteristics is of particular interest in the dermato-cosmetic industry, and this has been fuelled by the interest of the general public for naturally derived products. Observed enzyme inhibitory properties of *N. baytopii* extracts might be explained by their chemical components. Some components such as apigenin, naringenin, luteolin, and chlorogenic acid have been reported as significant enzymes inhibitors [44,45,46,47,48,49,50,51,52,53,54], and thus, *N. baytopii* could be considered as a promising source of natural enzyme inhibitors. Interestingly, several researchers reported on the enzyme inhibition abilities of some *Nepeta* species. For example, Sarikurkcu et al. [55] reported the inhibitory properties of *N. nuda* subsp. *glandulifera* and *N. cadmea* on cholinesterases, amylase, glucosidase, and tyrosinase. When compared with our results, the *Nepeta* species exhibited lower enzyme inhibition properties than *N. baytopii*. Furthermore, different research groups reported the enzyme-inhibitory effects of several *Nepeta* essential oils. For example, *N. nuda* and *N. cadmea* essential oils exhibited moderate inhibitory effects on some enzymes, and the main compounds were geijerene and nepetalactone in these essential oils, respectively [9]. As a structure-ability approach, essential oils have a complex nature, and thus, observed enzyme inhibitory abilities could be caused by different factors, including the main compounds and interactions of these components.

Several species of the *Lamiaceae* family have been studied for the development of novel chemotherapeutic agents [56,57,58]. Herein, the methanol and water extracts were tested with HepG2, human hepatocarcinoma cells (Table 6). Hepatocellular carcinoma, the most common liver malignancy, is a leading cause of cancer-related death worldwide [58]. In this work, the methanol extract of *N. baytopii* aerial parts showed high cytotoxicity (31.7%) against HepG2 while water was non-cytotoxic. Melanoma is a type of skin cancer occurring in melanocytes, which are dendritic-like cells producing melanin pigment [59]. We observed that the water extract (70.2%) showed higher cytotoxicity against mouse melanoma cell (B16 4A5). We also determined the cytotoxic effect of *N. baytopii* aerial parts against non-tumoral murine bone marrow stromal, and the results are presented in Table 7. The methanol extract (34.8%) was more cytotoxic than the water extract (61.5%). In the literature, in accordance with the presented results, several *Nepeta* species, such as *N. curvidens* [60], *N. curviflora* [61], and *N. nuda* [22] exhibited remarkable cytotoxic effects on several cell lines.

## 3. Materials and Methods

### 3.1. Plant Material

The aerial parts of *Nepeta baytopii* were collected in July 2019 (Genç village, Bingöl, Turkey, 38°43′00′′ N, 40°34′09′′ E, 1055 m). The plant material was authenticated by one of the authors (R.P). Voucher specimens (GP-1082) were deposited in the Bingöl University, Faculty of Agriculture, Bingöl, Turkey. Twenty-five plants were randomly collected in the same population, and they were dried in a dark condition for 10 days.

### 3.2. Extraction

The aerial parts of the plant materials were grounded, and then 10 g were separately extracted with hexane, ethyl acetate, methanol, and methanol/water (80%) in maceration technique (for 24 h, room temperature). The extracts were evaporated to dryness and stored at 4 °C until analysis. Regarding water extracts, we used traditional infusion techniques, and 5 g plant materials were kept with 100 mL of boiled water for 15 min. Then, the water extracts were lyophilised. The extracts procedure were performed in triplicate and the obtained extracts were stored at 4 °C until analysis.

### 3.3. UHPLC-MS Analysis

Chromatographic separation was accomplished with a Dionex Ultimate 3000RS UHPLC instrument, equipped with a Thermo Accucore C_18_ (100 mm × 2.1 mm i. d., 2.6 μm) analytical column for the separation of compounds. Water (A) and methanol (B) containing 0.1% formic acid were employed as mobile phases, respectively. The total run time was 70 min for the elution profile. Mass spectrum analysis was carried out using a Thermo Q-Exactive Orbitrap mass spectrometer (Thermo Scientific, Waltham, MA, USA) equipped with an electrospray ionisation probe interface in positive and negative-ion mode. All detailed analytical conditions have been published [62].

### 3.4. Essential Oil Components’ Analyses

The dried plant materials (100 g) were subjected to hydro-distillation using a Clevenger-type apparatus for 6 h. EO distillates, once yielded, were dried over anhydrous magnesium sulphate, filtered and then stored in dark bottles at −4 °C until further analysis. The yield was calculated as 0.52% (*v*/*w*).

The essential oil was analysed by gas chromatography-flame ionisation detector (GC-FID) and gas chromatography-mass spectrophotometry (GC-MS) techniques [63,64]. GC-MS analysis was conducted by an Agilent 5975 GC-MSD system coupled to an Agilent 7890A GC (Agilent Technologies Inc., Santa Clara, CA, USA). An HP-Innowax FSC column (60 m × 0.25 mm, 0.25 μm film thickness) was used with helium (purity 99.99%) as a carrier gas (1.2 mL/min). Other analytical details were reported in our previous papers [63,64]. The identification of components was based on a retention index (RI) determined by co-injection with reference to a homologous series of *n*-alkanes (C8–C30), under the same experimental conditions. Further identifications were achieved by comparing their mass spectra with those from NIST 05 and Wiley Eighth version, as well as by comparison of their RIs with literature values.

### 3.5. Total Phenolic and Flavonoid Content

Spectrophotometric methods were used to determine total phenolic and flavonoid content, as conducted previously. Standard equivalents (gallic acid equivalent (GAE) for phenolic and rutin equivalent (RE) for flavonoid) were used to assess the bioactive contents in the plant extracts [65,66].

### 3.6. Determination of Antioxidant and Enzyme-Inhibitory Effects

Antioxidant protocols included reducing power (cupric-reducing antioxidant capacity (CUPRAC) and ferric-reducing power (FRAP)), metal chelating, phosphomolybdenum (PBD), and free-radical scavenging (2,2-diphenyl-1-picrylhydrazyl (DPPH) and 3-ethylbenzothiazoline-6-sulphonic acid (ABTS)) activities. Experimental details were as described previously by [67]. Inhibitory effects of the extracts were tested against different enzymes (tyrosinase, α-amylase, α-glucosidase, and cholinesterases). Trolox and ethylenediaminetetraacetic acid (EDTA) for antioxidant, galantamine for cholinesterases, kojic acid for tyrosinase, and acarbose for α-amylase and α-glucosidase were used to express antioxidant and enzyme-inhibitory results.

### 3.7. Cell Culture

The human hepatocarcinoma HepG2 cells and murine bone marrow stromal S17 cells were kindly provided by the Centre for Molecular and Structural Biomedicine of Biomedical and Molecular BME, University of Algarve, Portugal), while mouse melanoma B16 4A5 cells were purchased from Sigma-Aldrich (Taufkirchen, Germany). All cell lines were cultured in Dulbecco’s Modified Eagle medium (DMEM), supplemented with foetal bovine serum (10%), L-glutamine (2 mM, 1%), and penicillin (50 U/mL)/streptomycin (50 μg/mL) (1%), and kept under a humidified atmosphere at 37 °C and 5% CO_2_.

### 3.8. Determination of Cellular Viability and Selectivity

Cells were plated in 96 well plates at 5 × 10^3^ cells/well (HepG2 and S17) and 2 × 10^3^ cells/well (B16 4A5). After a 24 h incubation period, cells were treated with the samples at the concentration of 100 μg/mL for 72 h. Cells incubated with DMSO at 0.5% (the highest DMSO concentration used in the test wells) were used as the control. The cellular viability was determined by the MTT 3-(4,5-dimethylthiazol-2-yl)-2,5-diphenyltetrazolium bromide) test, as described formerly [68]. The percentage of viable cells was calculated relative to the control (DMSO, 0.5%).

### 3.9. Statistical Analysis

All quantitative analyses were performed in triplicate (*n* = 3), and data were expressed as means ± S.D. Significant differences in the tested samples were determined by an ANOVA (Tukey test), with a probability value of 5%. Pearson’s correlation was estimated to identify the relationship between the total amounts of phenolics and flavonoids, and the biological activities (antioxidant and enzyme-inhibitory effects). R software (Version 3.6.2) was used for the statistical analysis.

## 4. Conclusions

For the first time, the biological activities and phytochemical profiles of the aerial parts of *N. baytopii,* endemic from Turkey, were evaluated. Extraction of the aerial parts was performed using solvents of different polarity. Furthermore, the essential oil of the plant was prepared by hydro-distillation. The water/methanol and water extracts possessed appreciable amounts of phenolic compounds and showed the highest antioxidant capacities in vitro. Phytochemical profiling revealed the presence of flavones, such as luteolin and apigenin and their derivatives in both the water and methanol extracts. The ethyl acetate extract showed pronounced inhibitory properties against butyrylcholinesterase, highlighting the possibility for a new, efficient Alzheimer’s disease therapeutic agent. The high cytotoxicity of *N. baytopii* aerial parts methanol extract against HepG2 suggests further future investigations in this area. The data presented here showed that the endemic *N. baytopii* possessed many interesting biological activities and is certainly encouraging for a future application in the pharmaceutical and nutraceutical fields, although further tests are necessary.

## Figures and Tables

**Table 1:** Total bioactive compounds and total antioxidant capacity (by phosphomolybdenum assay) of the tested extracts.

Extracts	TPC (mg GAE/g)	TFC (mg RE/g)
n-Hexane	13.23 ± 0.21 * ^e^	7.77 ± 0.07 ^e^
Ethyl acetate	19.57 ± 0.24 ^d^	27.02 ± 0.60 ^a^
Methanol	33.81 ± 0.22 ^c^	23.78 ± 0.87 ^b^
Water/methanol	41.25 ± 0.18 ^b^	10.61 ± 0.54 ^d^
Water	50.30 ± 0.13 ^a^	13.48 ± 0.18 ^c^
Essential oil	nt	nt

* Values are reported as mean ± SD. TPC: total phenolic content; TFC: total flavonoid content; GAE: gallic acid equivalent; RE: rutin equivalent; nt: not tested. Different letters indicate significant differences in the tested extracts (*p* < 0.05).

**Table 2:** Chemical composition of the methanol extract.

No.	Name	Formula	Rt	[M + H]^+^	[M − H]^−^	Fragment 1	Fragment 2	Fragment 3	Fragment 4	Fragment 5
1	Quinic acid	C_7_H_12_O_6_	1.23		191.06	173.04	171.03	127.04	93.03	85.03
2	Pantothenic acid	C_9_H_17_NO_5_	6.13	220.12		202.11	184.10	174.11	116.03	90.06
3	Caftaric acid (2-O-Caffeoyltartaric acid)	C_13_H_12_O_9_	8.54		311.04	179.03	149.01	135.04	87.01	
4	Neochlorogenic acid (5-O-Caffeoylquinic acid)	C_16_H_18_O_9_	10.11	355.10		163.04	145.03	135.04	117.03	89.04
5	Unidentified iridoid	C_16_H_24_O_9_	13.14		405.14	359.14	197.08	179.07	153.05	71.01
6	Salicylic acid-O-hexoside	C_13_H_16_O_8_	13.50		299.08	137.02	113.02	93.03	85.03	71.01
7	Mussaenosidic acid or isomer	C_16_H_24_O_10_	13.57		375.13	213.08	169.09	151.08	125.06	107.05
8	Kynurenic acid	C_10_H_7_NO_3_	13.80	190.05		162.06	144.04	116.05	89.04	
9 ^1^	Chlorogenic acid (3-O-Caffeoylquinic acid)	C_16_H_18_O_9_	14.84	355.10		163.04	145.03	135.04	117.03	89.04
10	Fertaric acid (2-O-Feruloyltartaric acid)	C_14_H_14_O_9_	14.85		325.06	193.05	178.03	149.06	134.04	87.01
11	Caffeic acid	C_9_H_8_O_4_	15.15		179.03	135.04	107.05			
12	Benzofuranecarbaldehyde	C_9_H_6_O_2_	15.53	147.04		119.05	91.05	65.04		
13	Cryptochlorogenic acid (4-O-Caffeoylquinic acid)	C_16_H_18_O_9_	16.08	355.10		163.04	145.03	135.04	117.03	89.04
14	5-O-(4-Coumaroyl)quinic acid	C_16_H_18_O_8_	17.40		337.09	191.06	173.04	163.04	119.05	93.03
15	4-O-(4-Coumaroyl)quinic acid	C_16_H_18_O_8_	18.03		337.09	191.06	173.04	163.04	119.05	93.03
16	Phaselic acid (2-O-Caffeoylmalic acid)	C_13_H_12_O_8_	18.62		295.05	179.03	135.04	133.01	115.00	71.01
17	Loliolide	C_11_H_16_O_3_	20.01	197.12		179.11	161.10	135.12	133.10	107.09
18	Eriodictyol-O-hexoside	C_21_H_22_O_11_	20.76		449.11	287.06	151.00	135.04	107.01	83.01
19	7-Deoxyloganic acid isomer	C_16_H_24_O_9_	20.95		359.13	197.08	153.09	135.08	109.06	89.02
20	Luteolin-O-hexosylglucuronide	C_27_H_28_O_17_	21.44		623.12	285.04	217.05	199.04	175.04	133.03
21	Luteolin-7-O-glucuronide	C_21_H_18_O_12_	22.75		461.07	285.04	217.05	199.04	175.04	133.03
22	Luteolin-7-O-glucoside (Cynaroside)	C_21_H_20_O_11_	22.86		447.09	327.05	285.04	284.03	256.04	151.00
23	Apigenin-O-hexosylglucuronide	C_27_H_28_O_16_	22.97		607.13	269.05	151.00	113.02		
24 ^1^	Apigenin-7-O-glucuronide	C_21_H_18_O_11_	24.52		445.08	269.05	175.02	151.00	113.02	
25	Rosmarinic acid (Labiatenic acid)	C_18_H_16_O_8_	24.71		359.08	197.05	179.03	161.02	135.04	72.99
26 ^1^	Eriodictyol (3′,4′,5,7-Tetrahydroxyflavanone)	C_15_H_12_O_6_	25.40		287.06	151.00	135.04	125.02	107.01	83.01
27	3-O-Methylrosmarinic acid	C_19_H_18_O_8_	26.58		373.09	197.05	179.03	175.04	160.02	135.04
28 ^1^	Naringenin (4′,5,7-Trihydroxyflavanone)	C_15_H_12_O_5_	27.72		271.06	227.07	177.02	151.00	119.05	107.01
29 ^1^	Luteolin (3′,4′,5,7-Tetrahydroxyflavone)	C_15_H_10_O_6_	28.37		285.04	217.05	199.04	175.04	151.00	133.03
30 ^1^	Apigenin (4′,5,7-Trihydroxyflavone)	C_15_H_10_O_5_	30.23		269.05	227.04	225.06	151.00	149.02	117.03
31	Dimethoxy-trihydroxy(iso)flavone	C_17_H_14_O_7_	30.38		329.07	314.04	313.04	299.02	271.03	
32	Dihydrololiolide	C_11_H_18_O_3_	30.51	199.13		181.12	163.11	135.12	111.04	107.09
33	Undecanedioic acid	C_11_H_20_O_4_	31.30		215.13	197.12	153.13	125.10	57.03	
34	Malyngic acid or isomer	C_18_H_32_O_5_	32.54		327.22	309.21	291.20	229.14	211.13	171.10
35	Nakhsmyrin or isomer	C_14_H_12_O_4_	32.67	245.08		227.07	217.09	203.07	175.04	
36	Nakhsmyrin or isomer	C_14_H_12_O_4_	33.29	245.08		227.07	217.09	203.07	175.04	
37	Dodecanedioic acid	C_12_H_22_O_4_	33.74		229.14	211.13	185.15	167.14		
38	Pinellic acid	C_18_H_34_O_5_	33.83		329.23	311.22	293.21	229.14	211.13	99.08
39	Caffeic acid phenethyl ester	C_17_H_16_O_4_	34.10		283.10	179.03	178.03	161.02	135.04	133.03
40	Salvigenin (5-Hydroxy-4′,6,7-trimethoxyflavone)	C_18_H_16_O_6_	35.33	329.10		314.08	313.07	296.07	285.08	268.07
41	Octadecenedioic acid	C_18_H_32_O_4_	37.99		311.22	293.21	235.17	223.17		
42	Stearidonic acid	C_18_H_28_O_2_	40.16		275.20	257.19	231.21	59.01		
43	Hydroxyoctadecatrienoic acid	C_18_H_30_O_3_	40.22		293.21	275.20	235.17	223.13	171.10	59.01
44	Stearidonic acid methyl ester	C_19_H_30_O_2_	42.15	291.23		259.21	241.20	217.20	107.09	93.07
45	Linoleamide	C_18_H_33_NO	44.44	280.26		263.24	245.23	109.10	95.09	81.07
46	Oleamide	C_18_H_35_NO	45.68	282.28		265.25	247.24	97.10	83.09	69.07

^1^ Confirmed by standards. Fragment: the fragments of compounds reflect a unique pattern in the mass spectrum.

**Table 3:** Chemical composition of the infusion.

No.	Name	Formula	Rt	[M + H]^+^	[M − H]^−^	Fragment 1	Fragment 2	Fragment 3	Fragment 4	Fragment 5
1	Quinic acid	C_7_H_12_O_6_	1.22		191.06	173.04	171.03	127.04	93.03	85.03
2	Pantothenic acid	C_9_H_17_NO_5_	6.16	220.12		202.11	184.10	174.11	116.03	90.06
3	Caftaric acid (2-O-Caffeoyltartaric acid)	C_13_H_12_O_9_	8.42		311.04	179.03	149.01	135.04	87.01	
4	Neochlorogenic acid (5-O-Caffeoylquinic acid)	C_16_H_18_O_9_	10.12	355.10		163.04	145.03	135.04	117.03	89.04
5	Unidentified iridoid	C_16_H_24_O_9_	13.10		405.14	359.14	197.08	179.07	153.05	71.01
6	Salicylic acid-O-hexoside	C_13_H_16_O_8_	13.48		299.08	137.02	113.02	93.03	85.03	71.01
7	Mussaenosidic acid or isomer	C_16_H_24_O_10_	13.54		375.13	213.08	169.09	151.08	125.06	107.05
8	Kynurenic acid	C_10_H_7_NO_3_	13.76	190.05		162.06	144.04	116.05	89.04	
9 ^1^	Chlorogenic acid (3-O-Caffeoylquinic acid)	C_16_H_18_O_9_	14.79	355.10		163.04	145.03	135.04	117.03	89.04
10	Fertaric acid (2-O-Feruloyltartaric acid)	C_14_H_14_O_9_	14.81		325.06	193.05	178.03	149.06	134.04	87.01
11	Benzofuranecarbaldehyde	C_9_H_6_O_2_	15.47	147.04		119.05	91.05	65.04		
12	Cryptochlorogenic acid (4-O-Caffeoylquinic acid)	C_16_H_18_O_9_	16.06	355.10		163.04	145.03	135.04	117.03	89.04
13	5-O-(4-Coumaroyl)quinic acid	C_16_H_18_O_8_	17.37		337.09	191.06	173.04	163.04	119.05	93.03
14	4-O-(4-Coumaroyl)quinic acid	C_16_H_18_O_8_	18.00		337.09	191.06	173.04	163.04	119.05	93.03
15	Loliolide	C_11_H_16_O_3_	19.98	197.12		179.11	161.10	135.12	133.10	107.09
16	Eriodictyol-O-glucuronide	C_21_H_20_O_12_	20.69		463.09	287.06	175.02	151.00	135.04	113.02
17	Eriodictyol-O-hexoside	C_21_H_22_O_11_	20.75		449.11	287.06	151.00	135.04	107.01	83.01
18	7-Deoxyloganic acid isomer	C_16_H_24_O_9_	20.94		359.13	197.08	153.09	135.08	109.06	89.02
19	Luteolin-O-hexosylglucuronide	C_27_H_28_O_17_	21.43		623.12	285.04	217.05	199.04	175.04	133.03
20	Luteolin-7-O-glucuronide	C_21_H_18_O_12_	22.74		461.07	285.04	217.05	199.04	175.04	133.03
21	Luteolin-7-O-glucoside (Cynaroside)	C_21_H_20_O_11_	22.85		447.09	327.05	285.04	284.03	256.04	151.00
22	Apigenin-O-hexosylglucuronide	C_27_H_28_O_16_	22.96		607.13	269.05	151.00	113.02		
23 ^1^	Apigenin-7-O-glucuronide	C_21_H_18_O_11_	24.51		445.08	269.05	175.02	151.00	113.02	
24	Rosmarinic acid (Labiatenic acid)	C_18_H_16_O_8_	24.71		359.08	197.05	179.03	161.02	135.04	72.99
25	N-trans-Feruloyltyramine	C_18_H_19_NO_4_	25.12	314.14		194.08	177.05	149.06	145.03	121.07
26 ^1^	Eriodictyol (3′,4′,5,7-Tetrahydroxyflavanone)	C_15_H_12_O_6_	25.40		287.06	151.00	135.04	125.02	107.01	83.01
27	3-O-Methylrosmarinic acid	C_19_H_18_O_8_	26.59		373.09	197.05	179.03	175.04	160.02	135.04
28 ^1^	Luteolin (3′,4′,5,7-Tetrahydroxyflavone)	C_15_H_10_O_6_	28.39		285.04	217.05	199.04	175.04	151.00	133.03
29 ^1^	Apigenin (4′,5,7-Trihydroxyflavone)	C_15_H_10_O_5_	30.23		269.05	227.04	225.06	151.00	149.02	117.03
30	Dihydrololiolide	C_11_H_18_O_3_	30.52	199.13		181.12	163.11	135.12	111.04	107.09
31	Undecanedioic acid	C_11_H_20_O_4_	31.32		215.13	197.12	153.13	125.10	57.03	
32	Malyngic acid or isomer	C_18_H_32_O_5_	32.55		327.22	309.21	291.20	229.14	211.13	171.10
33	Nakhsmyrin or isomer	C_14_H_12_O_4_	32.69	245.08		227.07	217.09	203.07	175.04	
34	Nakhsmyrin or isomer	C_14_H_12_O_4_	33.30	245.08		227.07	217.09	203.07	175.04	
35	Dodecanedioic acid	C_12_H_22_O_4_	33.76		229.14	211.13	185.15	167.14		
36	Pinellic acid	C_18_H_34_O_5_	33.85		329.23	311.22	293.21	229.14	211.13	99.08
37	Salvigenin (5-Hydroxy-4′,6,7-trimethoxyflavone)	C_18_H_16_O_6_	35.35	329.10		314.08	313.07	296.07	285.08	268.07
38	Octadecenedioic acid	C_18_H_32_O_4_	38.00		311.22	293.21	235.17	223.17		
39	Stearidonic acid	C_18_H_28_O_2_	40.19		275.20	257.19	231.21	59.01		
40	Hydroxyoctadecatrienoic acid	C1_8_H_30_O_3_	40.23		293.21	275.20	235.17	223.13	171.10	59.01
41	Stearidonic acid methyl ester	C_19_H_30_O_2_	42.15	291.23		259.21	241.20	217.19	107.09	93.07
42	Linoleamide	C1_8_H3_3_NO	44.45	280.26		263.24	245.23	109.10	95.09	81.07
43	Oleamide	C_18_H_35_NO	45.71	282.28		265.25	247.24	97.10	83.09	69.07

^1^ Confirmed by standards. Fragment: the fragments of compounds reflect a unique pattern in the mass spectrum.

**Table 4:** Chemical profile of the tested essential oil.

No	Compounds	RRI ^a^	(%)
1	Dihyroedulan I	1530	6.1
2	β-Bourbonene	1531	3.7
3	Linalool	1548	4.1
4	cis-p-mentha-2,8-dien-1-ol	1678	5.7
5	Verbonene	1732	2.1
6	(E)-β-Damascenone	1841	0.8
7	Caryophyllene oxide	2017	39.3
8	Hexahydrofarnesyl acetone	2134	2.7
9	Spathulenol	2147	15.6
10	n-Hexadecanoic acid	2912	11.0
	Total identified (%)		91.1

^a^ Relative retention indices are calculated against n-alkanes.

**Table 5:** Antioxidant properties of the tested extracts.

Extracts	PBD (mmol TE/g)	DPPH (mg TE/g)	ABTS (mg TE/g)	CUPRAC (mg TE/g)	FRAP (mg TE/g)	MCA (mg EDTAE/g)
n-Hexane	1.28 ± 0.15 ^c^	na	12.04 ± 0.84 ^d^	44.08 ± 0.35 ^e^	22.38 ± 0.66 ^e^	0.34 ± 0.02 ^e^
Ethyl acetate	2.36 ± 0.20 ^a^	na	13.33 ± 0.58 ^d^	75.55 ± 0.60 ^d^	29.24 ± 0.27 ^d^	22.15 ± 2.16 ^b^
Methanol	2.45 ± 0.15 ^a^	90.88 ± 0.37 ^c^	92.43 ± 1.30 ^b^	165.54 ± 1.87 ^c^	88.78 ± 1.36 ^c^	15.61 ± 0.54 ^c^
Water/methanol	1.87 ± 0.06 ^b^	94.40 ± 0.09 ^a^	129.22 ± 0.78 ^a^	221.71 ± 2.59 ^b^	124.78 ± 1.40 ^b^	26.88 ± 2.10 ^a^
Water	2.09 ± 0.07 ^a,b^	93.16 ± 0.20 ^b^	86.56 ± 2.54 ^c^	229.37 ± 1.38 ^a^	129.55 ± 1.23 ^a^	27.14 ± 0.58 ^a^
Essential oil	2.22 ± 0.15 ^a,b^	na	12.10 ± 0.53 ^d^	21.42 ± 0.11 ^f^	10.95 ± 0.13 ^f^	6.52 ± 0.07 ^d^

EDTAE: EDTA equivalents; PBD: phosphomolybdenum; TE: trolox equivalent; na: non active. Different letters indicate significant differences in the tested extracts (*p* < 0.05).

**Table 6:** Cellular viability (%) of *Nepeta baytopii* extracts on HepG2, B16 4A5 and S17 cell lines applied at the concentration of 100 µg/mL.

Sample/Cell line	HepG2	B16 4A5	S17
**DMSO 0.5%**	101 ± 7	88.2 ± 2.1	79.3 ± 4.9
**Methanol**	31.7 ± 0.5	76.7 ± 2.3	34.8 ± 0.9
**Water**	108.6 ± 12.6	70.2 ± 3.1	61.5 ± 5.6

**Table 7:** Enzyme inhibitory effects of the tested extracts.

Extracts	AChE (mg GALAE/g))	BChE (mg GALAE/g)	α-Amylase (mmol ACAE/g)	α-Glucosidase (mmol ACAE/g)	Tyrosinase (mg KAE/g)
n-Hexane	3.97 ± 0.32 ^b^	6.93 ± 1.14 ^b^	0.66 ± 0.01 ^b^	7.87 ± 0.02 ^b^	77.84 ± 1.83 ^b^
Ethyl acetate	4.57 ± 0.06 ^a^	10.85 ± 0.73 ^a^	0.84 ± 0.02 ^a^	7.76 ± 0.01 ^b^	78.60 ± 1.58 ^b^
Methanol	3.65 ± 0.11 ^b^	2.98 ± 0.46 ^c^	0.67 ± 0.02 ^b^	8.15 ± 0.08 ^a^	96.06 ± 0.70 ^a^
Water/methanol	2.68 ± 0.07 ^c^	na	0.50 ± 0.01 ^c^	0.61 ± 0.04 ^e^	95.31 ± 1.77 ^a^
Water	na	na	0.10 ± 0.01 ^e^	1.06 ± 0.09 ^d^	6.15 ± 1.02 ^d^
Essential oil	na	na	0.24 ± 0.01 ^d^	1.64 ± 0.01 ^c^	21.41 ± 3.57 ^c^

GALAE: galantamine equivalent; KAE: kojic acid equivalent; ACAE: acarbose equivalent; na: non-active. Different letters indicate significant differences in the tested extracts (*p* < 0.05).

## Data Availability

Not applicable.

## Supplementary Materials

The following are available online at https://www.mdpi.com/article/10.3390/plants10061176/s1, Figure S1. Total ion chromatograms of methanol extract in positive ion mode (a) and negative ion mode (b). Figure S2. Total ion chromatograms of water extract in positive ion mode (a) and negative ion mode (b).
