# Supplementary material for: Chemical Profiling and Biological Evaluation of Nepeta baytopii Extracts and Essential Oil: An Endemic Plant from Turkey
Source: Plants (Basel). 2021 Jun 9;10(6):1176. doi: 10.3390/plants10061176 (PMC8228258; doi:10.3390/plants10061176)
Supplement: Supplementary file 1 [file plants-10-01176-s001.zip › plants-1250848-supplementary.pdf]

Type of the Paper (Article)

Chemical Profiling and Biological Evaluation of *Nepeta baytopii* Extracts and Essential Oil: An Endemic Plant from Turkey

Gokhan Zengin[1], Mohamad Fawzi Mahomoodally[2], Abdurrahman Aktumsek[1], József Jekő[3], Zoltán Cziáky[3], Maria João Rodrigues[4], Luisa Custodio[4], Rıdvan Polat[5], Ugur Cakilcioglu[6], Adnan Ayna[7], Monica Gallo[8*], Domenico Montesano[9*], Carene Picot-Allain[2]

[1]Physiology and Biochemistry Research Laboratory, Department of Biology, Science Faculty, Selcuk University, Campus, 42130 Konya, Turkey. Email: gokhanzengin@selcuk.edu.tr (G.Z.) and aktumsek@selcuk.edu.tr (A.A.)
[2]Department of Health Sciences, Faculty of Medicine and Health Sciences, University of Mauritius, Réduit 230, Mauritius. Email: f.mahomoodally@uom.ac.mu (M.F.M.) and picotcarene@yahoo.com (C.P.)
[3]Agricultural and Molecular Research and Service Institute, University of Nyíregyháza, Nyíregyháza, Hungary. Email: cziaky.zoltan@nye.hu (C.Z.) and jjozsi@gmail.com (J.J.)
[4] Centre of Marine Sciences, University of Algarve, Faculty of Sciences and Technology, Ed. 7, Campus of Gambelas, 8005-139 Faro, Portugal. Email: mary_p@sapo.pt (M.J.R) and lcustodio@ualg.pt (L.C.)
[5]Department of Landscape Architecture, Faculty of Agriculture, Bingol University, 12000, Bingöl. Turkey. Email: rpolat@bingol.edu.tr (R.P.)
[6]Munzur University, Pertek Sakine Genç Vocational School, Tunceli, Turkey. Email: ucakilcioglu@yahoo.com (U.C.)
[7]Department of Chemistry, Faculty of Sciences and Arts, Bingol University, 12000, Bingöl, Turkey. Email: aayna@bingol.edu.tr (A.Ay.)
[8]Department of Molecular Medicine and Medical Biotechnology, University of Naples Federico II, via Pansini, 5, 80131 Naples, Italy E-mail: mongallo@unina.it (M.G.)
[9]Department of Pharmacy, University of Naples Federico II, via D. Montesano 49, 80131 Naples, Italy. E-mail: domenico.montesano@unina.it (D.M.)

* Correspondence: domenico.montesano@unina.it (D.M.); mongallo@unina.it (M.G.).

Citation: Zengin, G.; Mahomoodally, M.F.; Aktumsek, A.; Jekő, J.; Cziáky, Z.; Rodrigues, M.J.; Custodio, L.; Polat, R.; Cakilcioglu, U.; Ayna, A.; et al. Chemical Profiling and Biological Evaluation of Nepeta baytopii Extracts and Essential Oil: An Endemic Plant from Turkey. *Int. J. Mol. Sci.* **2021**, *10*, 1176. https://doi.org/10.3390/plants10061176

Academic Editor: Petko Denev

Received: 22 May 2021
Accepted: 7 June 2021
Published: 9 June 2021

Publisher's Note: MDPI stays neutral with regard to jurisdictional claims in published maps and institutional affiliations.

Copyright: © 2021 by the authors. Submitted for possible open access publication under the terms and conditions of the Creative Commons Attribution (CC BY) license (https://creativecommons.org/licenses/by/4.0/).

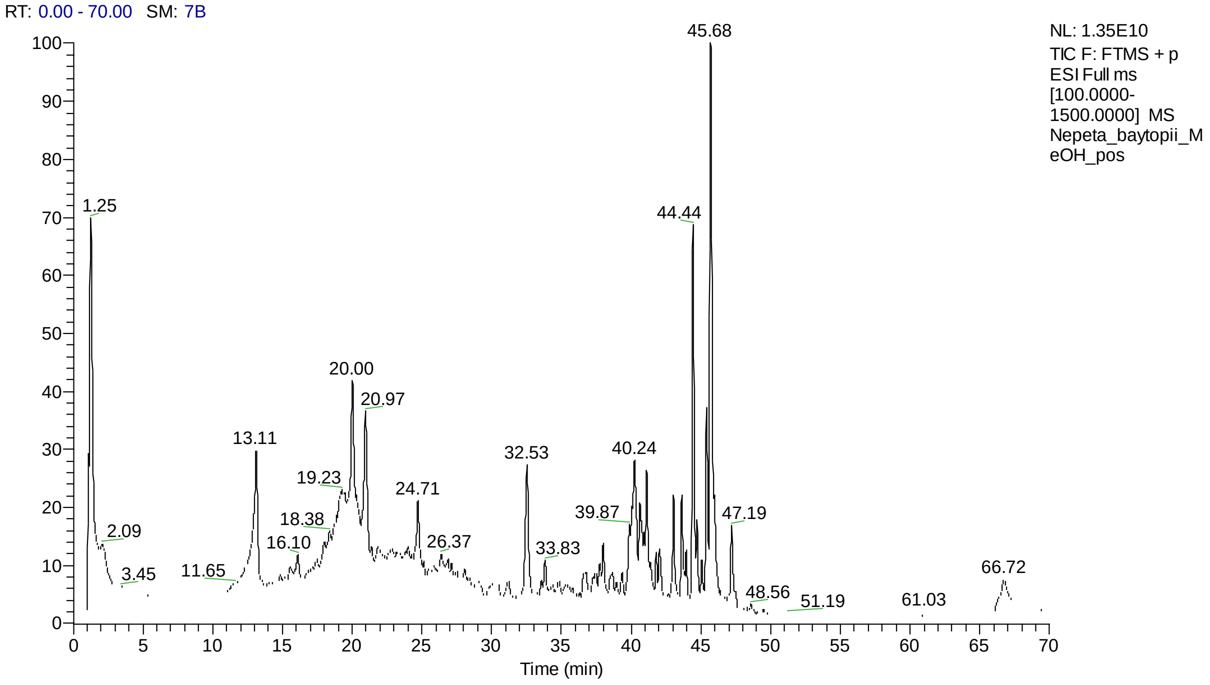

(a)

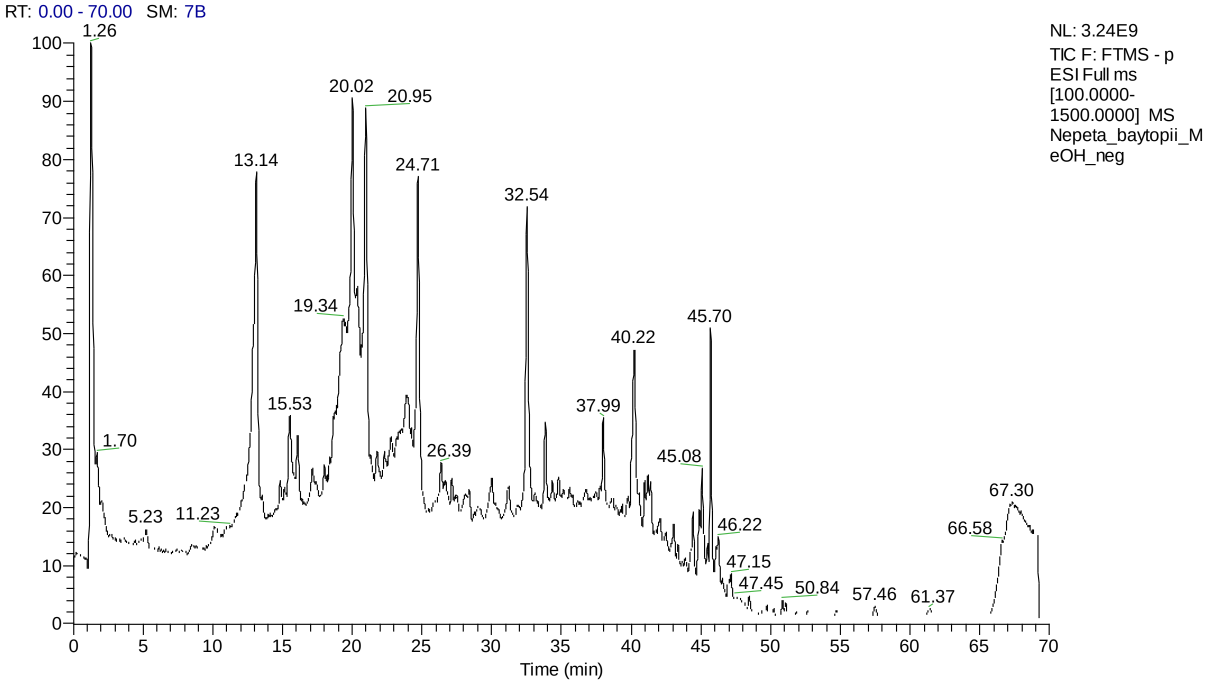

(b)

Figure S1. Total ion chromatograms of methanol extract in positive ion mode (a) and negative ion mode (b)

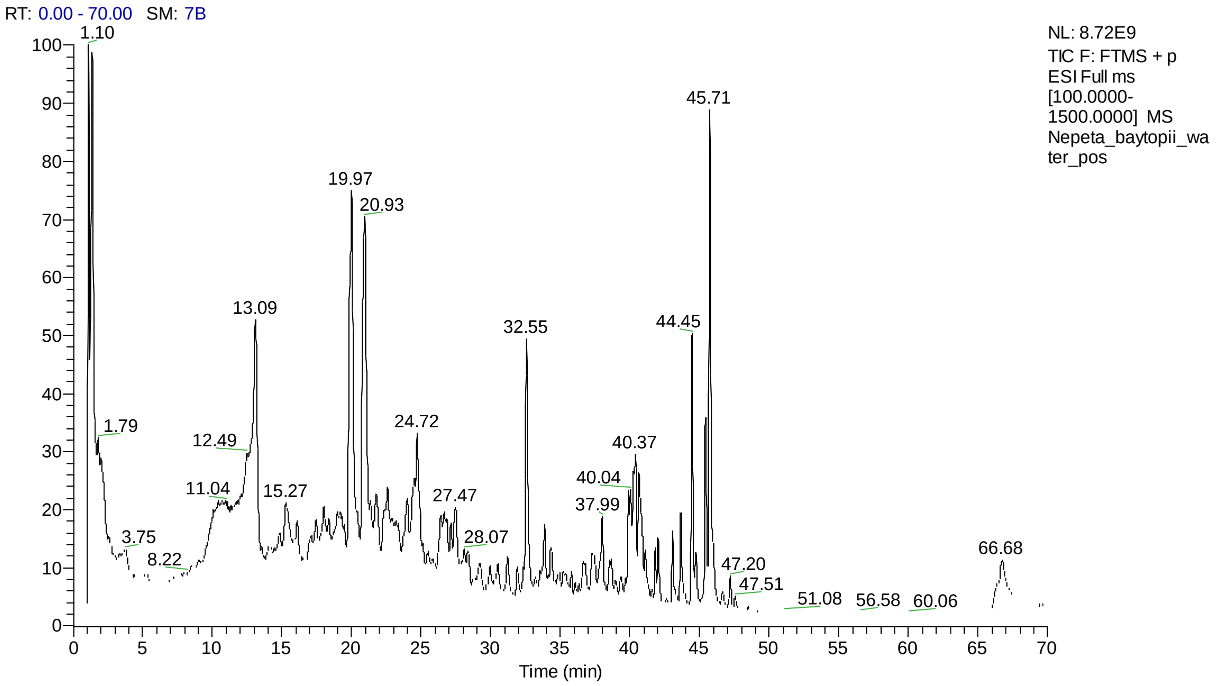

(a)

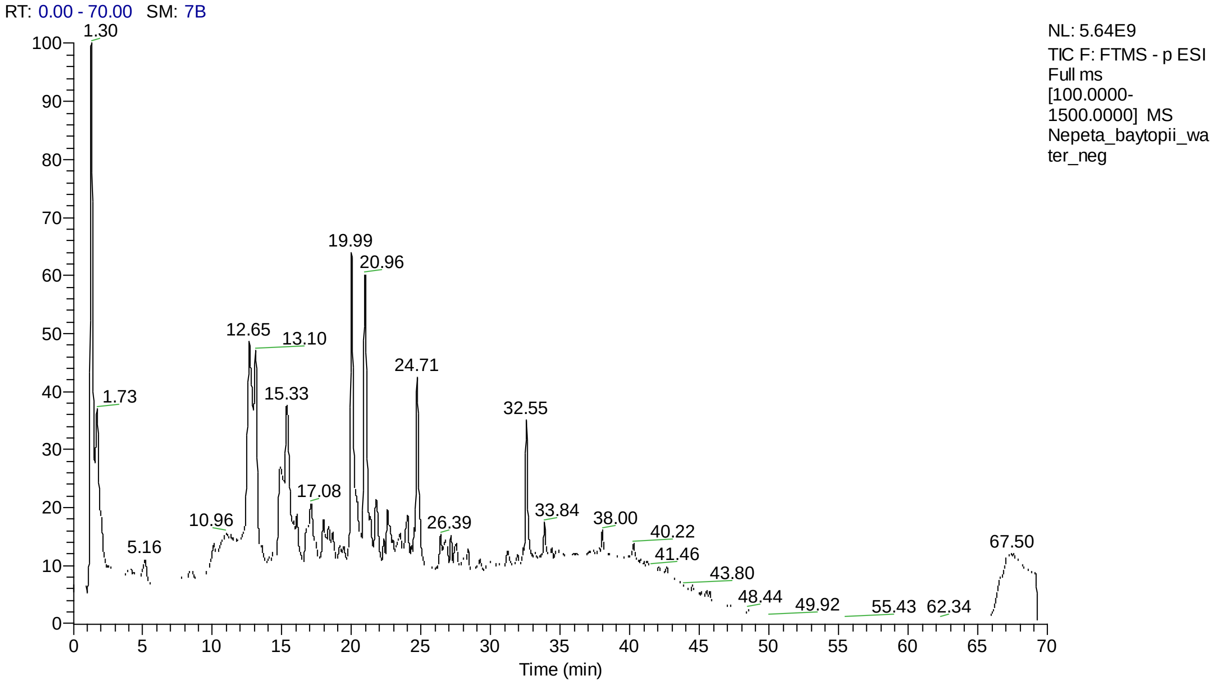

(b)

Figure S2. Total ion chromatograms of water extract in positive ion mode (a) and negative ion mode (b)
